# Femtosecond near-infrared laser microirradiation reveals a crucial role for PARP signaling on factor assemblies at DNA damage sites

**DOI:** 10.1093/nar/gkv976

**Published:** 2015-09-30

**Authors:** Gladys Mae Saquilabon Cruz, Xiangduo Kong, Bárbara Alcaraz Silva, Nima Khatibzadeh, Ryan Thai, Michael W. Berns, Kyoko Yokomori

**Affiliations:** 1 Beckman Laser Institute and Medical Clinic, University of California, Irvine, 1002 Health Sciences Road East, Irvine, CA 92612, USA; 2 Department of Biological Chemistry, School of Medicine, University of California, Irvine, CA 92697–1700, USA; 3 Department of Developmental and Cell Biology, School of Biological Sciences, University of California, Irvine, CA 92617, USA; 4 Department of Biomedical Engineering and Surgery, University of California, Irvine, CA 92617, USA

## Abstract

Laser microirradiation is a powerful tool for real-time single-cell analysis of the DNA damage response (DDR). It is often found, however, that factor recruitment or modification profiles vary depending on the laser system employed. This is likely due to an incomplete understanding of how laser conditions/dosages affect the amounts and types of damage and the DDR. We compared different irradiation conditions using a femtosecond near-infrared laser and found distinct damage site recruitment thresholds for 53BP1 and TRF2 correlating with the dose-dependent increase of strand breaks and damage complexity. Low input-power microirradiation that induces relatively simple strand breaks led to robust recruitment of 53BP1 but not TRF2. In contrast, increased strand breaks with complex damage including crosslinking and base damage generated by high input-power microirradiation resulted in TRF2 recruitment to damage sites with no 53BP1 clustering. We found that poly(ADP-ribose) polymerase (PARP) activation distinguishes between the two damage states and that PARP activation is essential for rapid TRF2 recruitment while suppressing 53BP1 accumulation at damage sites. Thus, our results reveal that careful titration of laser irradiation conditions allows induction of varying amounts and complexities of DNA damage that are gauged by differential PARP activation regulating protein assembly at the damage site.

## INTRODUCTION

Genome integrity is continually threatened by reactive oxygen species generated during normal cellular respiration and by exposure to exogenous DNA damaging agents. The resulting DNA lesions, if left unrepaired, can accumulate mutations and/or cause chromosomal rearrangements/loss that can lead to cancer, developmental abnormalities and cell death. DNA double-strand breaks (DSBs) are the most deleterious type of DNA damage, which are recognized by specific DSB signaling and repair factors (1). Laser microirradiation can induce DNA damage at a specific submicron region in the cell nucleus, and has become a standard technique to study the DSB site recruitment or modifications of various factors *in vivo* (2–6). However, laser microirradiation often induces a mixture of different types and amounts of DNA damage depending on the irradiation conditions. Despite the efforts to compare different laser systems with each other, and with conventional damaging agents (e.g. γ irradiation and genotoxic chemicals) (5,7–10), how variable laser conditions/dosages affect the amounts and types of DNA damage and how they affect DNA damage response (DDR) have not been fully determined. As a result, recruitment or modification of several repair factors demonstrated using one laser system was found to be not reproducible by another system (5,7,11). Thus, it is pertinent to address the relationship between different laser irradiation conditions and DNA damage/DDR induction.

In the current study, we specifically addressed two such controversies, the recruitment of p53-binding protein 1 (53BP1 or TP53BP1) and telomeric repeat binding factor 2 (TRF2). 53BP1 plays a significant role in DSB signaling and is involved in DSB repair pathway choice (12–14). 53BP1 promotes the non-homologous end joining (NHEJ) repair pathway by inhibiting the DNA end-resection necessary for the homologous recombination (HR) pathway of DSB repair (15–19). 53BP1 is recruited to DNA damage sites through its focus-forming region (a.a. 1220–1711) that contains the oligomerization domain, the Tudor domain, and the ubiquitylation-dependent recruitment (UDR) motif (20–22). The Tudor domain recognizes methylated histone H4 lysine 20 (K20) residue and the UDR specifically binds to the ubiquitylated K15 residue of histone H2A. Previously it was found that high-dose ultraviolet A (UVA) laser-induced damage failed to effectively recruit 53BP1 despite the induction of high density DSBs and efficient recruitment of the NHEJ factor Ku (7). However, the reason for this failed recruitment of 53BP1 was unclear.

TRF2 is a telomere binding protein critical for telomere end protection (23–25). It binds directly to duplex telomeric (TTAGGG) repeats, stabilizes the T-loop structure, and prevents the activation of the DDR pathway by suppressing ataxia-telangiectasia-mutated (ATM) protein kinase (24,26–28). Previous studies also provided evidence that TRF2 is recruited to non-telomeric DNA damage sites and promotes DSB repair though its exact role in the process remains unclear (11,29–32). While depletion of TRF2 impairs HR repair (32), TRF2 phosphorylation by ATM appears to be important for NHEJ (31). Although TRF2 is recruited rapidly and transiently to high-irradiance laser-induced DNA lesions, TRF2 recruitment was not observed at damage sites induced by low-irradiance UV radiation or ionizing radiation despite the presence of DSBs in both cases (11,29,30). It remained unclear whether the failure to detect TRF2 was simply due to the low number of DSBs present at the damage site, or if it reflected qualitative differences of damage types and/or DDR induced by different systems.

We investigated the mechanisms underlying the differential recruitment of 53BP1 and TRF2 by varying laser microirradiation conditions using *Potorous tridactylus* (PtK) 2 cells as a primary model system. PtK2 cells have been used to study DDR and repair with laser microirradiation and imaging because the cell has a large nucleus and fewer chromosomes (33–35). Human cells were also used for comparison. The controlled site-specific laser microirradiation experiments were carried out using two different near-infrared (NIR) femtosecond (fs) laser systems. Their biological effects were examined over a range of *in situ* laser energy and peak irradiances. We defined the thresholds and ranges of laser energy dose/peak irradiance optimal for the recruitment of 53BP1 and TRF2, and found that their recruitment is critically regulated by differential activation of poly(ADP-ribosyl)ation (PAR) response. Poly(ADP-ribose) polymerases (PARPs) are activated by DNA damage and is involved in base excision repair (BER) as well as single-strand break (SSB) and DSB repair (36). Recent studies also demonstrated the significance of PAR at the damage sites in the recruitment of chromatin modifiers that facilitate DSB repair, suggesting the critical scaffolding role of PAR modification at damage sites (2). Our results provide strong evidence that well-controlled laser microirradiation is highly valuable in determining the correlation between different amounts and complexity of DNA damage, DDR signaling and the behavior of individual DNA repair factors as well as the choice of repair pathway *in vivo*. Our results reveal that DNA damage is gauged by differential PARP activation that dictates repair factor assembly at damage sites.

## MATERIALS AND METHODS

### Cell lines, plasmids and cell culture

PtK2 kidney epithelial cells (American Type Culture Collection ATCC, CCL 56), stably expressing TRF2-AID-YFP (37) or EGFP-53BP1^1220-1711^ were generated and grown as previously described (38). For TRF2-AID-YFP, no auxin was used for the experiments, and thus, the fusion protein is referred to as TRF2–YFP in the rest of the study. The mammalian expression plasmids for GFP–TRF2 and GFP–NTH1 were transiently transfected using lipofectamine 2000 (Life Technologies). Cells were damaged and examined at around 24 h after transfection. HeLa cells were grown in Dulbecco's modified Eagle's medium (DMEM; Gibco) supplemented with L-Glutamine, 10% fetal bovine serum (FBS) and antibiotics. HT1080 human fibrosarcoma cells were grown in DMEM with high glucose (4500 mg/l), supplemented with 10% (v/v) FBS, 100 units/ml penicillin. Cells were grown at 37°C with 5% CO_2_. For laser microirradiation and immunofluorescence experiments, cells were trypsinized (TrypLE^TM^ Express, Life Technologies) and plated on 35 mm gridded imaging dishes (MatTek) at approximately 2 × 10^4^ cells per dish. The media were replaced before laser microirradiation with Hanks’ Balanced Salt Solution (HBSS, 1X) to avoid absorption of the laser light by the phenol red. For the endogenous TRF2 detection, cells were incubated with 10 ng/ml Hoechst 33258 (Sigma) for 30 min (min), and were washed with MEM media twice before irradiation.

### Laser microirradiation

Laser microirradiation was performed using two different laser systems. The RoboLase ablation software was used to control irradiation from a mode-locked Ti:Sapphire NIR pulsed femtosecond laser (Mira-900, Coherent Inc.) tuned at 800 nm wavelength. The pulse width and repetition rate of the Mira-900 laser system are 200 fs and 76 MHz, respectively. Technical specifications of the Mira-900 laser system are summarized in Table 1. The laser beam was expanded, collimated, and steered through a series of mirrors and lenses and was coupled into the side port of a motorized inverted microscope (Zeiss Axiovert, 200 M). The laser beam was focused by a phase contrast, oil immersion, 63X/1.4 NA objective (Zeiss, Plan-Apochromat, Ph3) to a diffraction-limited focal spot with a calculated diameter of ≈697 nm. A dual-axis (XY) fast steering mirror (FSM, 200–01, Newport Corp.) was placed in the beam path before the microscope in order to enable scanning the laser focal point across the sample plane. The laser energy and irradiance at the specimen (*in situ*) were controlled by varying the orientation of a Glan–Thompson laser polarizer introduced in the beam path and mounted on a computer-controlled motorized rotational stage (PR50PP, Newport Corp.). The duration of the laser microirradiation was controlled by gating the laser beam using an electromechanical laser shutter (Uniblitz, Vincent Associates). Prior to each experiment, the laser power entering the microscope side port, i.e. ‘the input power’ (referred to throughout this manuscript), was measured and is given as mW. This was followed by measurement of the laser power at the back aperture of the objective by removing the objective from the microscope turret and allowing an unobstructed laser beam to illuminate a 19 mm diameter sensor of a FieldMaxII-TOP power meter coupled to a PowerMax PM3 probe (Coherent Inc., Santa Clara). The *in situ* laser power (the power in the focal spot in the cell) was calculated by multiplying the power entering the back aperture of the objective by the objective transmission at 800 nm. The transmission coefficient of the objective at 800 nm was determined to be ≈47% based on a three objective measurement method (39). The *in situ* laser energy per pulse and the peak irradiances were calculated based on the calculated values of the *in situ* laser powers at each laser input power. A range of laser input powers was generated through the computerized controlled rotary movement of the Glan–Thompson laser polarizer. Values of the measured laser input powers along with the corresponding calculated values of the *in situ* energy per laser pulse and peak irradiances in the focal spot are presented in Table 2 for the Mira-900 laser system. As shown in Table 2, the values of the *in situ* energy per laser pulse and peak irradiance were in the range of ≈5.33 × 10^−2^ to 4.13 × 10^−1^ nJ and ≈7 × 10^10^ to 5.41 × 10^11^ W/cm^2^, respectively. Cells were monitored via fluorescence microscopy using a Zeiss inverted microscope (Axiovert 200 M) equipped with a Hamamatsu Orca cooled CCD Camera (C10600–10B-H, Hamamatsu Photonics, Japan).

**Table 1.** Comparison of the specific parameters of Mira-900 and Meta fs laser systems used in our studies for laser microirradiation experiments

Parameters	Mira-900 System Ti: Sapphire fs Laser Coherent Inc.	LSM 510 Meta System Ti: Sapphire fs Laser Chameleon-Ultra, Coherent Inc.
Wavelength (nm)	800	780
Pulse width (fs)	200	140
Repetition rate (MHz)	76	80
Objective parameters	63X/1.4 NA	100X/1.3 NA
Diffraction limited diameter of laser at focal (nm)	697	732

**Table 2.** Laser microirradiation parameters and the associated biological observations over a range of input powers examined in our study with Mira-900 laser system

Input laser power (mW)*	*In situ* energy per pulse (nJ)	*In situ* peak irradiance (W/cm^2^)
20	5.33 × 10^−2^	6.99 × 10^10^
25	6.67 × 10^−2^	8.74 × 10^10^
60	1.6 × 10^−1^	2.1 × 10^11^
85	2.27 × 10^−1^	2.97 × 10^11^
95	2.53 × 10^−1^	3.31 × 10^11^
100	2.67 × 10^−1^	3.49 × 10^11^
125	3.33 × 10^−1^	4.36 × 10^11^
155	4.13 × 10^−1^	5.41 × 10^11^

*This measurement is made on the laser beam prior to entry into the microscope. The subsequent columns in this table representing laser energy and irradiance that are calculated by determining the laser power entering the back aperture of the objective and then using the three objective method (see previous discussion in Materials and Methods section) determining the transmission through the objective.

Laser microirradiation and DNA damage experiments were also performed with a Zeiss LSM 510 META NLO laser-scanning microscope system (Meta system) (Table 1). The system contains a Zeiss Axiovert 200 M microscope and combines standard fluorescence confocal imaging at six different excitation wavelengths with multi-photon fluorescence/second harmonic generation. A mode-locked Ti:Sapphire pulsed fs laser (Chameleon Ultra, Coherent Inc.) tunable in a wavelength range of 690 to 1040 nm was coupled to the microscope as the irradiation source. The laser wavelength, pulse width, and repetition rate of the Meta system are 780 nm, 140 fs and 80 MHz, respectively. Technical specifications of the Meta laser system are provided in Table 1. The scan rate (pixel dwell time) of the laser excitation source is 12.8 μs per pixel. Similar to the Mira-900 system, the laser beam was collimated, expanded, steered by a series of mirrors into the microscope, and passed through an objective (100X/1.3 NA) to a diffraction limited spot with a calculated diameter of ≈732 nm. The laser power was controlled by changing the laser power transmission percent parameter through the user interface software provided by the company. Similar to the experiments with the Mira-900 laser system, the laser beam power at the back aperture of the objective was measured prior to each laser microirradiation experiment, and the corresponding *in situ* laser power was calculated based on the objective transmission at 780 nm. Values of *in situ* energy per laser pulse and peak irradiances were calculated and are shown in Table 3.

**Table 3.** Microirradiation energy and power density calculation in our experiments with LSM 510 Meta laser system

Power transmission parameter (%)	Energy per pulse at focal (nJ)	Peak irradiance at focal (W/cm^2^)
15	1.91 × 10^−2^	3.24 × 10^10^
20	2.49 × 10^−2^	4.23 × 10^10^
25	3.10 × 10^−2^	5.27 × 10^10^

### Antibodies and immunofluorescent staining

Cells were fixed with 4.0% paraformaldehyde-tris-buffer saline (TBS) for 10 min at room temperature (RT), permeabilized with 0.5% Triton X-100 for 5 min at 4°C, and placed on ice. Immunofluorescent staining was performed as previously described (38,40). The following primary antibodies were used: mouse monoclonal antibodies specific for γH2AX (05–636, Millipore), PAR polymers (BML-SA216–0100, Enzo Life Sciences, Inc.), ubiquitin (Ub) (spa-205, StressGen), TRF2 (NB100–56506, Novus Biologicals), XRCC1 (GTX72311, Gene Tex, Inc.), ATM (GTX70103, Gene Tex, Inc.), DNA–PKcs (ab1832, Abcam), 53BP1 (MAB3802, Millipore), and cyclobutane pyrimidine dimer (CPD) (MC-062, Kamiya Biomedical Company) as well as rabbit polyclonal antibodies specific for γH2AX (07–164; Millipore), PAR (4336-BPC-100, Trevigen), XPA (GTX100112, Gene Tex, Inc.), CtIP (ab70163, Abcam), 53BP1 (sc-22760, Santa Cruz Biotech., Inc.), phosphor-Chk1 Ser-345 (2348, Cell Signaling), phosphor-Chk2 Thr-68 (2661, Cell Signaling) and MDC1 (NB100–395, Novus Biologicals). Affinity-purified rabbit anti-PARP1 antibody was previously described (41). After incubation, cells were washed twice in PBS/0.05% Tween-20 for 5 min at RT, and incubated with secondary antibodies (Invitrogen; 1:1000) for 1 h at RT. Cell images were acquired using a Hamamatsu digital CCD Camera (ORCA-R2, C10600) coupled to Zeiss Axiovert, 200 M with a 63X/1.4 NA objective (Zeiss, Plan-Apochromat, oil, Ph3), CCD camera (Olympus, FVII) coupled to Olympus Olympus IX81 with a 100X objective (UPlanFI, oil Ph3, NA 1.3), or Zeiss LSM510 META confocal microscope with a 100X objective (Plan-Neofluor, oil, Ph3, NA 1.3). Images were analyzed using ImageJ software (NIH, Bethesda, MD).

### TUNEL assay

TUNEL staining of PtK2 cells were performed essentially as described (38) using the TUNEL label mix (Roche Applied Science). For positive control, fixed and permeabilized cells were incubated with DNase I (3000 U/ml in 50 mM Tris-HCl, pH 7.5, 10 mM MgCl_2_ 1 mg/ml BSA) for 10 min at room temperature prior to labeling procedures.

### Inhibitors of PARP, ATM, DNA-PK and PARG

Inhibitors (PARP inhibitor (Pi) 100 μM NU1025 (Sigma) or 20 μM olaparib (Apexbio Technology), 10 μM DNA–PKcs inhibitor (Di) NU7026 (Sigma), 10 μM ATM inhibitor (Ai) KU55933 (Calbiochem), 1 μM PARG inhibitor (PARGi) DEA ((6,9-diamino-2-ethoxyacridine lactate monohydrate) (Trevigen)) were added to the cell culture one hour prior to damage induction. DMSO only was added to control cells.

### SiRNA transfection

Control and PARP siRNA transfections were performed as previously described (42). TRF2 siRNA (SI00742630, Qiagen) was also used.

## RESULTS

### Microirradiation energy, irradiance, and cell viability analyses for the Mira-900 and Meta laser systems

The fs NIR laser systems have been used to induce DSBs and to study DDRs in both human and marsupial cells, including PtK2 cells (5,38,40,43). In order to examine the effects of varying laser microirradiation conditions on 53BP1 and TRF2 recruitment, the experiments were performed in a range of *in situ* laser energy and peak irradiances in PtK2 cells. For this purpose, the laser power entering the microscope (input power) as well as at the objective back aperture, and the *in situ* laser power at the focal spot were controlled using a laser polarizer (see the Materials and Methods section). The values of the corresponding *in situ* laser energy per pulse and peak irradiances were calculated at each input power, and are shown in Table 2 for the Mira-900 laser system. The results obtained with the Meta laser system were also compared (Tables 1 and 3). Cell viability was confirmed at 7–8 h post irradiation (p.i.) at the different laser irradiation conditions (Supplemental Figure S1A). Twenty cells were further monitored and were all found to be viable at 24–31 h following irradiation (data not shown). The TUNEL assay indicated that DNA strand breaks were confined to the laser-irradiated regions at 5 min p.i. and decreased after 1 h suggesting ongoing repair (Supplemental Figure S1B).

### Differential recruitment of 53BP1 and TRF2 to low and high input-power damage sites

**Figure 1.**
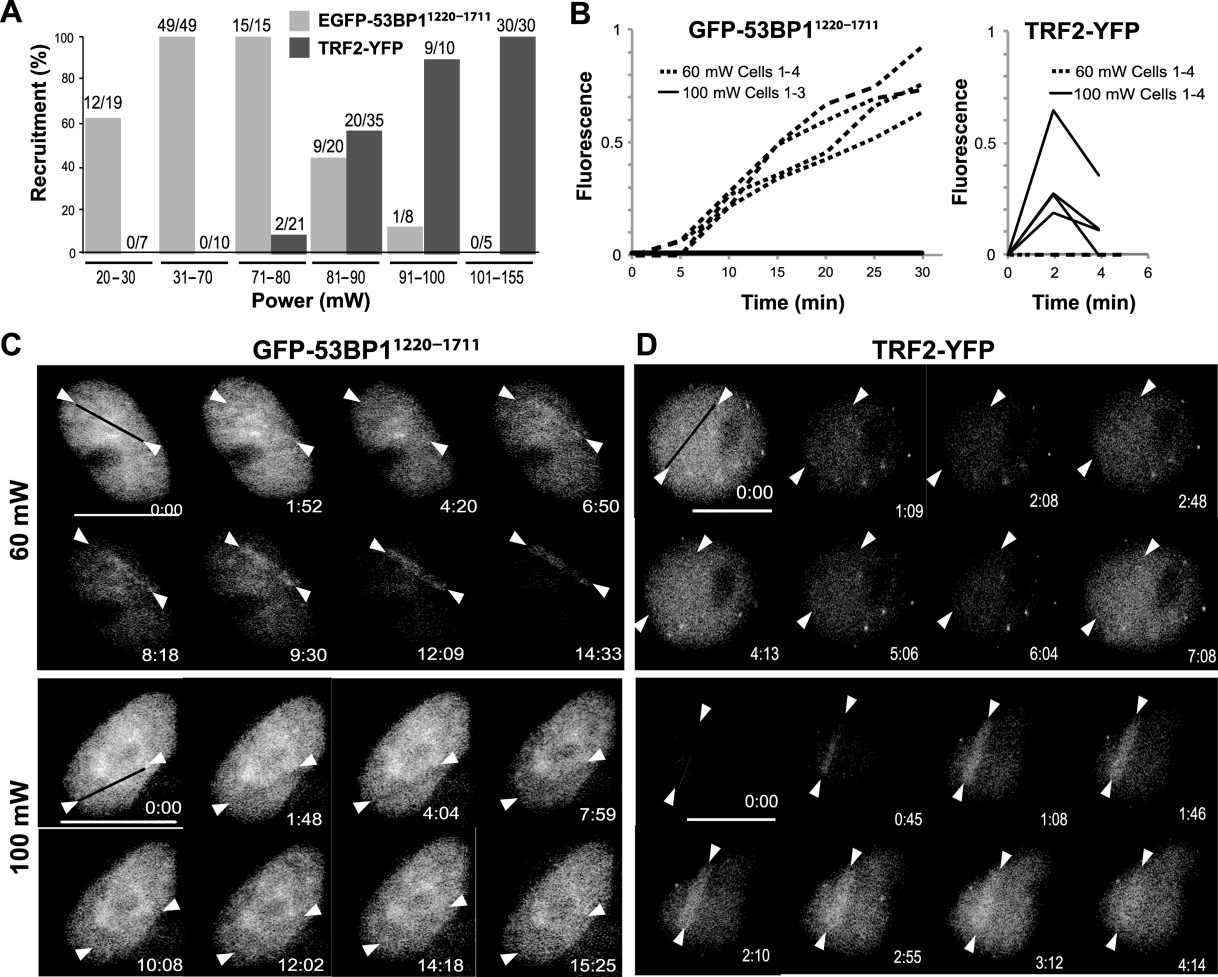
Recruitment of 53BP1 and TRF2 to fs NIR laser-induced DNA lesions. (**A**) EGFP-53BP1^1220-1711^ and TRF2-YFP cell lines were subjected to NIR laser microirradiation with the input power ranging from 20 mW to 155 mW (corresponding to ≈7 × 10^10^ W/cm^2^ to 5.41 × 10^11^ W/cm^2^ peak irradiance) as indicated. EGFP-53BP1^1220-1711^ and TRF2-YFP recruitment was monitored for 15 min and 6 min p.i., respectively. The number of the cells with the recruitment and the number of cells examined are shown at the top of each histogram. Detailed microirradiation energy and irradiance calculations versus the laser input power (the power of the laser beam entering the microscope) are shown in Table 2. (**B**) Time course fluorescence measurement of the damage-site recruitment of EGFP-53BP1^1220-1711^ and TRF2-YFP. PtK2 cells stably expressing EGFP-53BP1^1220-1711^ or TRF2-YFP were damaged with either low (60 mW input power with peak irradiance of ≈2.1 × 10^11^ W/cm^2^; upper panel) or high (100 mW input power with peak irradiance of 3.49 × 10^11^ W/cm^2^; lower panel) dose of NIR laser microirradiation as indicated. For EGFP-53BP1^1220-1711^, four and three cells were followed for 30 min for 60 mW and 100 mW damage, respectively. For TRF2-YFP, four cells each were followed for 4 min. The fluorescent intensity at the damage site was divided by that in the nucleoplasm and was subtracted by one for normalization. (**C**) Similar experiments as in (**B**) were performed following EGFP-53BP1^1220-1711^ recruitment, and live fluorescent images of one representative cell each at indicated p.i. time points are shown. Time 0:00 refers to pre-laser condition. The irradiation site is indicated by a black line at time 0:00 and white arrowheads. Consistent results were obtained with 12 and 8 cells examined for the low (60 mW) and high (100 mW) irradiation condition, respectively. Scale bar = 10 μm. (**D**) Similar experiments as in (**C**) for TRF2-YFP. Consistent results were obtained with 11 and 12 cells examined for the low (60 mW) and high (100 mW) irradiation condition, respectively. Scale bar = 10 μm.

Using PtK2 cell lines stably expressing either EGFP-53BP1^1220-1711^ or TRF2-YFP, we examined the recruitment of these proteins to laser-induced damage sites. EGFP-53BP1^1220-1711^ was shown to faithfully recapitulate the damage site recruitment of the full-length 53BP1 (20–22), and TRF2-YFP was shown to be functional in telomere targeting (37). We found that the low threshold detection limit of EGFP-53BP1^1220-1711^ was 25 mW (6.67 × 10^−2^ nJ *in situ* energy per 200 fs pulse, 8.74 × 10^10^ W/cm^2^ *in situ* peak irradiance); the high threshold detection was 85 mW (2.27 × 10^−1^ nJ, 2.97 × 10^11^ W/cm^2^) (Figure 1A). In contrast, the low threshold detection limit of TRF2-YFP was at an input power of 85 mW. We defined the threshold as the power at which EGFP-53BP1^1220-1711^ or TRF2-YFP was detectable in >50% of the damaged cells during the first 15 min or 6 min p.i., respectively. The TRF2-YFP recruitment was observed in 100% of the cells even at the highest irradiation condition examined (4.13 × 10^−1^ nJ, 5.41 × 10^11^ W/cm^2^, corresponding to 155 mW of input power) (Figure 1A). The results reveal a clear difference in the optimal laser damage conditions for 53BP1 and TRF2. 53BP1 accumulates at the low-irradiance damage sites while TRF2 requires damage induced at higher irradiance.

Based on the above results, we chose two sets of input powers and peak irradiances for recruitment kinetic analyses: ‘low’ (60 mW input power, 1.6 × 10^−1^ nJ at 2.1 × 10^11^ W/cm^2^) and ‘high’ (100 mW input power, 2.67 × 10^−1^ nJ at 3.49 × 10^11^ W/cm^2^). With 60 mW, EGFP-53BP1^1220-1711^ recruitment was observed in 100% of the cells while no recruitment was detected at 100 mW over 15 min time period following laser irradiation (Figure 1A). With 60 mW, the recruitment of EGFP-53BP1^1220-1711^ became detectable at 5–7 min p.i., and the signal intensity kept increasing during the first 30 min following the damage (Figure 1B and Supplemental Figure S2A). With 100 mW, no significant recruitment of 53BP1^1220-1711^ was observed during the first 30 min p.i. (Figure 1B, left). Representative fluorescent live cell images for 15 min p.i. are shown (Figure 1C). EGFP-53BP1^1220-1711^ persisted at damage sites up to 24 h p.i. at 60 mW while delayed clustering was observed at 100 mW (Supplemental Figure S2A). More detailed time course analyses revealed weak and transient, if any, recruitment of EGFP-53BP1^1220-1711^ at 25 mW, compared to the robust accumulation at 60 mW damage sites that peaked around 3–4 h p.i. followed by a modest decrease even after 26 h p.i.(∼70% retained) (Supplemental Figure S3). Similar to 100 mW (Supplemental Figure S2A), weak and delayed accumulation of EGFP-53BP1^1220-1711^ was observed at damage sites induced by 125 mW input power after 4 h and eventually in 100% of cells at 26 h p.i. (Supplemental Figure S3). Similar tendencies were observed with the endogenous 53BP1 (Supplemental Figure S2B and C) though the endogenous 53BP1 can be detected more readily at earlier time points than EGFP-53BP1^1220-1711^ (Supplemental Figure S2C). This may be due to the contribution of other domains of 53BP1 that may facilitate the damage site recruitment. The results indicate that damage induced by high input power has an inhibitory effect on damage recognition by 53BP1.

In contrast to the attenuation of the 53BP1 recruitment, TRF2-YFP was clustered at the damage sites within one min p.i. in 100% of the cells damaged at 100 mW whereas no recruitment was observed at 60 mW damage sites (Figure 1A). TRF2-YFP recruitment is mostly limited to the first five min following 100 mW damage induction (Figure 1B, right). Representative fluorescent live cell images are shown (Figure 1D). Similar results were obtained with the endogenous TRF2 (Supplemental Figure S4). Visualization of the endogenous TRF2 at damage sites required photosensitization with Hoechst 33258 dye as described previously, which is possibly due to the low level of the endogenous TRF2 and low sensitivity of the antibody (11,29,30,44). Nevertheless, the recruitment of the endogenous TRF2 to damage sites in the presence of Hoechst was laser input-power-dependent similar to that of TRF2-YFP in the absence of Hoechst (Supplemental Figure S4). Addition of Hoechst had no significant effect on XRCC1 or PAR signals at damage sites, but specifically increased γH2AX signal, suggesting that the increase of DSBs correlates with the enhanced TRF2 recruitment (Supplemental Figure S5). Similar results were obtained using the Meta system at 15% (low) and 25% (high) input power (Supplemental Figure S6). Taken together, the results demonstrate differential damage site recruitment of 53BP1 and TRF2 *in vivo*.

### High input power induces complex DNA damage

**Figure 2.**
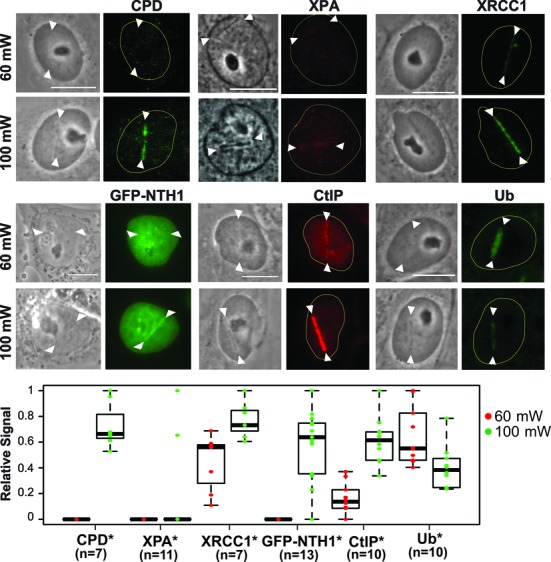
Characterization of damage induced by low and high input power laser microirradiation. Interphase PtK2 cells were irradiated at 60 mW and 100 mW input powers, corresponding to ≈2.1 × 10^11^ W/cm^2^ and ≈3.49 × 10^11^ W/cm^2^ peak irradiances, respectively. Cells were fixed and stained with antibody specific for CPD (N = 7 each for 60 mW and 100 mW; cells were fixed at 10 min p.i.), XPA (N = 11 each; cells were fixed at 3 min p.i.), and the SSB repair protein XRCC1 (N = 7 each; cells were fixed at 5 min p.i.). PtK2 cells expressing GFP-NTH1 were also irradiated at 60 mW and 100 mW input powers and were followed for 1 min (N = 13 each), Scale bar = 10 μm. Immunofluorescent detection of the DSB end-resection factor CtIP and ubiquitin (Ub) at 60 mW and 100 mW damage sites at 30 min p.i. (N = 10 each) was also performed. Representative images (including the live cell images of GFP-NTH1) are shown for the factors indicated at the top. Quantitative fluorescent intensity measurements of the damage-site recruitment were done as in Figure 1(B) and were displayed relative to the highest signal observed within in each group underneath. Asterisks confirm the significant *P*-values (<0.05) for the differences of the factor recruitment between 60 mW and 100 mW.

To better understand the DNA lesions induced under low and high input-power laser irradiation conditions, we performed immunofluorescent staining of endogenous DDR/repair proteins and protein modifications. We found that CPD were detectable at 100 mW (but not 60 mW) damage sites at 10 min p.i. (Figure 2). Induction of CPD by NIR was reported previously (5). Weak localization of the nuclear excision repair (NER) factor xeroderma pigmentosum group A (XPA) was detected in 2 out of 11 cells at 100 mW damage sites but none at 60 mW damage sites (Figure 2). Attenuated NER factor response compared to that at UVC-induced damage has been reported (10,45). XRCC1, which is involved in BER, SSB repair, and alternative NHEJ repair of DSBs was detectable at both, but stronger at 100 mW damage sites (Figure 2). We previously demonstrated that base damage specifically occurred at the damaged sites induced by high irradiation with the NIR laser (42). Consistent with this, the significant clustering of DNA glycosylase NTH1 fused to GFP was observed at 100 mW, but not 60 mW, damage sites, indicating the induction of base damage by high-, but not low-, irradiance laser damage (42) (Figure 2). This may also explain the increased XRCC1 signal, in addition to increased strand breaks, at 100 mW damage sites compared to 60 mW (Figure 2). The fluorescent signal for CtIP, which mediates DNA end resection important for the HR repair of DSBs, was also stronger at 100 mW than at 60 mW damage sites suggesting the increased DSBs (Figure 2). Taken together, the results indicate that the input power of 100 mW induces increased strand breaks as well as complex DNA damage containing crosslinking and base damage compared to 60 mW.

### Differential activation of DDR kinases and PARP signaling in response to low and high-input-power laser damage

**Figure 3.**
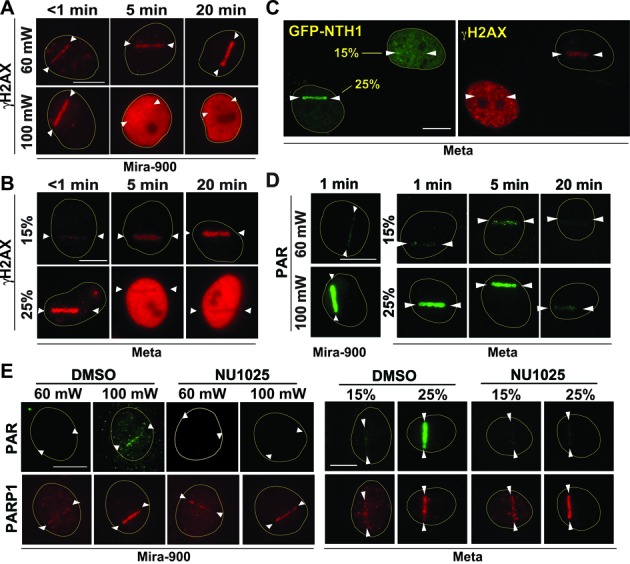
Comparison of phosphorylation of H2AX and PAR accumulation in response to low and high-input-power damage in two NIR systems. (**A**) Time course analysis of γH2AX following 60 mW and 100 mW damage in the Mira-900 system. PtK2 cells were fixed at specific time points p.i. as indicated at the top. Scale bar = 10 μm. (**B**) Similar analysis as in (**A**) with 15% and 25% input laser using the Meta system. Scale bar = 10 μm. (**C**) Localization of GFP-NTH1 DNA glycosylase and γH2AX response to laser damage with the indicated input power (15% and 25%) in the Meta system. Left: live cell image of GFP-NTH1 at 3 min p.i. Right: γH2AX immunostaining of the same cells fixed at 10 min p.i. Scale bar = 10 μm. (**D**) Left: Immunofluorescent staining of PAR at low (60 mW) and high (100 mW) input power in the Mira-900 system at 1 min p.i. Right: time course analysis of PAR response at low (15%) and high (25%) input power in the Meta system. Time points p.i. are indicated at the top. Scale bar = 10 μm. (**E**) Immunofluorescent staining of PAR and PARP1 at low and high input power at 3 min p.i. Mira-900 system (left) and Meta system (right) with and without PARP inhibitor NU1025. Scale bar = 10 μm.

It was recently reported that clustered damage by ionizing radiation causes pan-nuclear γH2AX in DDR kinases (ATM and DNA–PK)-dependent manner (46). This spreading was shown to be dependent on the amount of DNA damage (46). Similarly, we found that damage induced by the NIR laser with 100 mW input power also causes spreading of γH2AX to the whole nucleus (Figure 3A). Similar spreading of γH2AX was observed using high (25%), but not low (15%), input powers using the Meta system (Figure 3B). At 25% power, the significant clustering of GFP-NTH1, an indicator of base damage, was also observed similar to 100 mW in the Mira-900 system (Figures 2 and 3C; Supplemental Figure S6). Since this indicates the presence of base damage (42), we also examined the activation of PARP. We found that the PAR response is induced significantly by high input-power laser, but only weakly by low input-power laser, in both Mira-900 and Meta systems (Figure 3D). PAR signals, but not PARP1 protein localization, at damage sites were sensitive to the PARP inhibitor NU1025 or olaparib (Figure 3E and Supplemental Figure S9) (47,48). Taken together, the results reveal that the low and high input powers (irradiances) in the two NIR systems studied induce different degrees of DDR kinase activation and PARP response correlating with the number of DSBs and the complexity of the DNA damage.

### ATM/DNA–PK and PARP activation inhibits 53BP1 recruitment to damage sites

**Figure 4.**
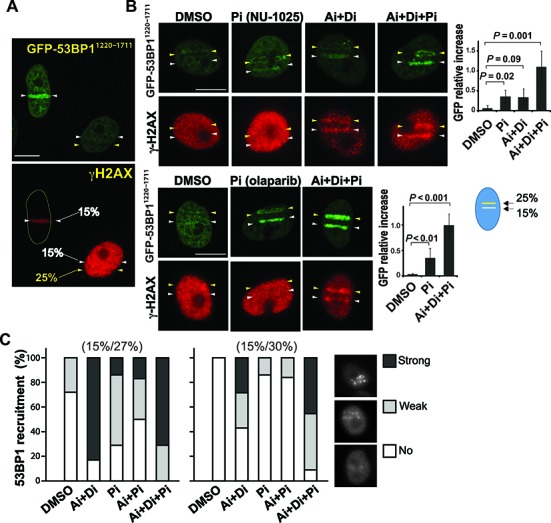
The effects of ATM/DNA–PK and PARP on 53BP1 recruitment to damage sites. (**A**) The presence of high input power damage inhibits the recruitment of 53BP1 to a low input power damage sites. PtK2 cells stably expressing EGFP-53BP1^1220-1711^ were microirradiated with single 15% input power (white arrowheads) or both 15% and 25% input power (white and yellow arrowheads, respectively) in the same nucleus using the Meta system. Live-cell imaging of EGFP-53BP1^1220-1711^ was captured at 30 min after DSB induction. Then cells were fixed and stained with antibody specific for γH2AX as a DSB marker. Scale bar = 10 μm. (**B**) A similar analysis as the right cell in (**A**). EGFP-53BP1^1220-1711^ PtK2 cells were irradiated with both 15% and 25% input power in the presence of DMSO, two different PARP inhibitors (Pi) (NU-1025 and olaparib as indicated), ATM and DNA-PK inhibitors (Ai+Di) and the combination of ATM, DNA–PK and PARP inhibitors (Ai+Di+Pi). Right: the EGFP-53BP1^1220-1711^ recruitment signal at the 15% input power-induced DNA damage sites in the presence of DMSO or different combinations of inhibitors was measured (see the schematic diagram). N = 10 for each treatment. *P*-values are shown. (**C**) Similar experiments in HeLa cells detecting the endogenous 53BP1. Pi (NU-1025) was used. One hour after DNA damage induction, cells were fixed and immunostained with antibody specific for 53BP1 to visualize the endogenous 53BP1. Results with two different high-input-power damage (27% on the left, and 30% on the right) in combination with 15% input-power damage are shown. The percentages of cells with ‘no’, ‘weak’, or ‘strong’ 53BP1 recruitment at DNA damage sites (representative images are shown) were quantified. N = 20 for each condition.

The above results raised the possibility that the presence of complex DNA damage and/or higher degrees of DDR kinase/PARP responses might have caused differential recruitment of 53BP1 and TRF2. Interestingly, the 53BP1^1220-1711^ recruitment to a low-power damage site was inhibited by presence of the second damage site induced by high-input-power in the same cell nucleus (Figure 4A). The results indicate that high-input-power damage suppresses 53BP1 recruitment *in trans*, suggesting that damage signaling induced by the high-irradiance damage is interfering with the recruitment of 53BP1. Thus, we treated damaged cells with ATM and DNA–PK inhibitors (Ai and Di, respectively) and PARP inhibitor (NU1025 or olaparib) (Pi) (Figure 4B). We found that Ai and Di effectively suppressed pan-nuclear γH2AX consistent with the recent study (46). This treatment partially restored clustering of 53BP1^1220-1711^ to both low- and high-input-power damage sites, indicating that ATM/DNA–PK hyperactivation had an inhibitory effect on 53BP1 recruitment. Interestingly, Pi (both NU1025 and olaparib) also partially restored 53BP1^1220-1711^ recruitment to damage sites. When cells were treated with both Ai+Di and Pi, efficient accumulation of 53BP1^1220-1711^ was observed at both low and high input-power damage sites within the same cell nucleus. The efficient recruitment of 53BP1 to high input-power damage sites in the presence of these inhibitors indicate that damage signaling induced by the high-irradiance damage, rather than the nature of the damage itself, was interfering with the recruitment of 53BP1. Similar results were obtained with the endogenous 53BP1 in HeLa cells treated with Ai, Di and/or Pi (Figure 4C). Thus, the results are neither unique to PtK2 cells nor recombinant fusion protein-specific.

**Figure 5.**
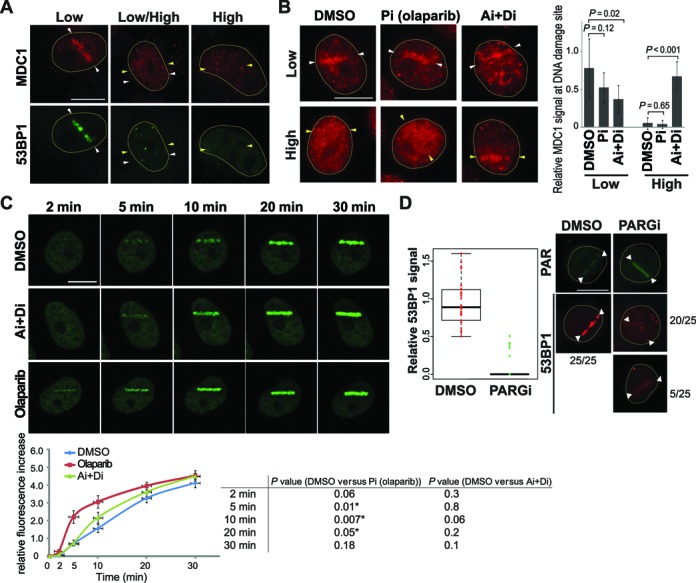
PARP activity affects 53BP1 recruitment to low-input power damage. (**A**) Immunofluorescent localization of MDC1 and 53BP1 in HeLa cells irradiated with 15% (indicated by white arrowheads) and/or 30% (indicated by yellow arrowheads) at 30 min p.i. N = 10 with consistent results. Scale bar = 10 μm. (**B**) The effect of Pi and Ai+Di treatment on MDC1 localization. The experiments were carried out as in (**A**) in the presence or absence of inhibitors indicated. Scale bar = 10 μm. Right: Fluorescent signals of MDC1 at low or high input-power damage sites in the presence of DMSO, Pi, or Ai+Di were measured. N = 10 for each treatment. *P*-values are shown. (**C**) The effect of Pi (olaparib) on the recruitment of EGFP-53BP1^1220-1711^ in response to low input-power damage. PtK2 cells expressing EGFP-53BP1^1220-1711^ were damaged by 15% input-power in the Meta system and were followed for 30 min as indicated (N = 8 for each treatment). Scale bar = 10 μm. Quantification of relative fluorescent signal increase for each treatment is shown underneath with corresponding *P*-values (asterisks indicate *P*-values < 0.05). (**D**) The effect of enhancement of PAR signals by PARG inhibitor (PARGi) treatment on the endogenous 53BP1 recruitment to low input-power damage sites. Left: quantitative analysis of endogenous 53BP1 recruitment with low input-power laser (60 mW) at 15 min post irradiation in the presence of DMSO or PARGi as indicated. Right: PAR and 53BP1 immunostaining pictures of cells damaged under the same conditions with DMSO or PARGi treatment. For DMSO treatment, 100% of cells examined (N = 25) exhibited robust 53BP1 recruitment (left). In contrast, only 5 out of 25 cells showed discernable 53BP1 recruitment in the presence of PARGi (bottom right), and the 53BP1 signals were weaker compared to DMSO. While 20 out of 25 showed no 53BP1 recruitment. Scale bar = 10 μm.

Spreading of γH2AX by clustered ionizing irradiation was shown to result in dispersion of MDC1, which directly binds to γH2AX, from the damage sites (46). We also observed displacement of MDC1 from high-input power damage sites (Figure 5A), which was restored by Ai+Di treatment that reduces γH2AX spreading (Figure 5B). MDC1 facilitates the 53BP1 accumulation at the damage sites by recruiting the Ub ligase RNF8 (49–51). This is consistent with the weaker Ub signal at 100 mW damage sites compared to 60 mW (Figure 2). Since 53BP1 recruitment is dependent on ubiquitylation of histone H2AK15 (20), low Ub at damage sites may contribute to decreased 53BP1 recruitment to damage sites. We also examined the localization of ATM and DNA–PK as well as phosphorylated Chk1 (pChk1) and Chk2 (pChk2) (Supplemental Figure S7). While ATM was recruited to both low and high input-power damage sites, DNA–PK appears to spread to the whole nucleus in the presence of high input-power damage (Supplemental Figure S7A and B). We found that pChk1 and pChk2 were robustly induced by high input-power damage. While pChk1 induction was primarily restricted to the high input-power damage site, pChk2 was diffuse throughout the nucleus as noted previously (52) in the presence of high input-power damage, and was effectively inhibited by the Ai+Di treatment (Supplemental Figure S7C and D). Thus, it is formally possible that Chk2 may also play a role in trans-inhibition of 53BP1 recruitment.

The Pi treatments had no effect on γH2AX spreading and MDC1 dispersion, suggesting that PARP-dependent inhibition of 53BP1 recruitment is mediated by a distinct mechanism (Figures 4B and 5B). Thus, the effect of PARP signaling on 53BP1 recruitment was further evaluated using the low input-power irradiation condition, in which pan-nuclear γH2AX does not occur (Figures 3B and 4A). We found that Pi significantly enhanced 53BP1 recruitment to low input-power damage sites while Ai and Di had a minimal effect (Figure 5C and Supplemental Figure S8). The enhancement effect was most prominently observed during the early phase of 53BP1 recruitment (∼first 20 min) correlating with the rapid and transient nature of PAR signaling. We also treated PtK2 cells with Poly(ADP-ribose) glycohydrolase (PARG) inhibitor (PARGi), DEA. Since the PAR chains are degraded by PARG, inhibition of PARG should increase the PAR signal at damage sites. At 15 min p.i. with the low input-power laser, the PAR signal was indeed enhanced significantly by DEA treatment, and we observed substantial reduction of the endogenous 53BP1 clustering at damage sites (Figure 5D). Thus, the results indicate that PARP signaling regulates the immediate early 53BP1 recruitment. Taken together, the results reveal that damage signaling critically determines 53BP1 recruitment to laser-induced damage sites.

### PARP activity is required for the rapid and transient recruitment of TRF2 to damage sites

**Figure 6.**
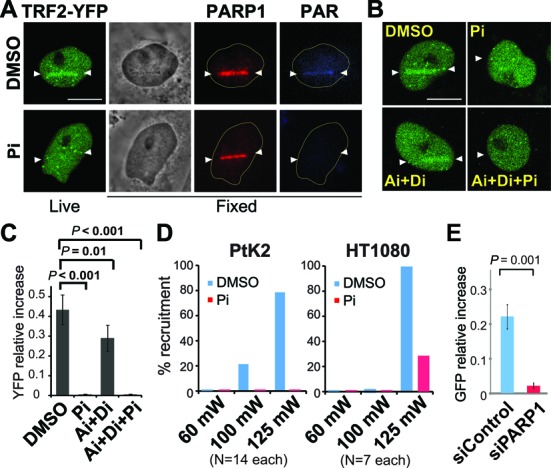
TRF2 is recruited to damage sites in a PARP activity-dependent manner. (**A**) PARPi abolished TRF2-YFP recruitment to damage sites. PtK2 cells stably expressing TRF2-YFP were treated with DMSO or PARP inhibitor (Pi) (NU1025) and damaged with 25% input Meta laser power. Live image of TRF2-YFP was taken at 1–2 min post irradiation at the peak of its recruitment. Cells were fixed at 15 min p.i. and co-stained with antibodies specific for PARP1 and PAR. Scale bar = 10 μm. (**B**) ATM and DNA–PK inhibitors (Ai and Di) had no significant effect on the recruitment of TRF2-YFP to damage sites. Similar experiments as in (**A**) but cells were treated with different combinations of Ai, Di and Pi as indicated. Scale bar = 10 μm. (**C**) The YFP signals at damage sites in (**B**) were measured before and after damage induction. Relative increase of the YFP signal was calculated as (YFP peak value after damage − YFP value before damage) / YFP value before damage. For control and PARPi-treated cells N = 15. For Ai+Di and Ai+Di+Pi, N = 6 each. (**D**) The recruitment of the endogenous TRF2 in DMSO or Pi-treated PtK2 (left) and human HT1080 (right) cells. Laser damage with indicated input powers was carried out in the presence of Hoechst 33258 dye as previously described (11,29,30). Fourteen PtK2 cells and seven HT1080 cells were damaged in each group (DMSO or Pi-treated) at each dosage. Cells were fixed at 1–2 min p.i. and were subjected to immunofluorescent staining using antibody specific for TRF2. Percentages of cells positive for TRF2 recruitment are shown. (**E**) Fluorescent measurement of GFP-TRF2 at 1 min p.i. in HeLa cells treated with control (blue) or PARP (red) siRNA.

The mechanism of TRF2 recruitment to DNA damage sites is not understood. Interestingly, we found that treatment of PtK2 cells with Pi (NU1025 or olaparib) significantly compromised accumulation of TRF2-YFP at high-input-power damage sites, indicating that damage site targeting of TRF2 is PARP-dependent (Figure 6A and B; Supplemental Figure S9). This is consistent with the fact that TRF2 is preferentially recruited to high-input-power damage sites that preferentially induce the PAR response, and may also explain the previous observations of recruitment of TRF2 only to high-irradiance laser damage (11,29,30). In agreement with a previous report (30), and unlike 53BP1, the Ai+Di treatment exhibited only a minor effect on TRF2 recruitment (Figure 6B and C). The endogenous TRF2 was equally sensitive to PARP inhibition in both PtK2 and human HT1080 cells (Figure 6D). Taken together, the results indicate that PARP signaling, but not ATM/DNA–PK signaling, mediates rapid and transient recruitment of TRF2 to damage sites.

Several PARP family members, PARP1, PARP2 and PARP3, play a role in DNA repair (36). Among them, PARP1 plays a major role in PAR accumulation at laser-induced damage sites (42). Depletion of PARP1 by siRNA was sufficient to suppress GFP-TRF2 recruitment to damage sites in HeLa cells, further confirming that PARP1 is responsible for the major PARP activity at damage sites (Figure 6E). Although TRF2 was reported to interact with PARP1 (53), Pi treatment that did not affect PARP1 localization at damage sites abolished TRF2 recruitment, indicating that physical interaction with PARP1 is not sufficient for damage site recruitment of TRF2 (Figure 6A and Supplemental Figure S9). TRF2 promotes the recruitment of PARP1 to eroded telomeres, but not vice versa (53,54). Depletion of TRF2, however, had no effect on PARP1 recruitment to damage sites (Supplemental Figure S10). Taken together, our results demonstrate that PARP signaling plays a critical role in the rapid TRF2 recruitment to DNA damage sites, which is distinct from the mechanism of TRF2 recruitment to telomeres.

## DISCUSSION

**Figure 7.**
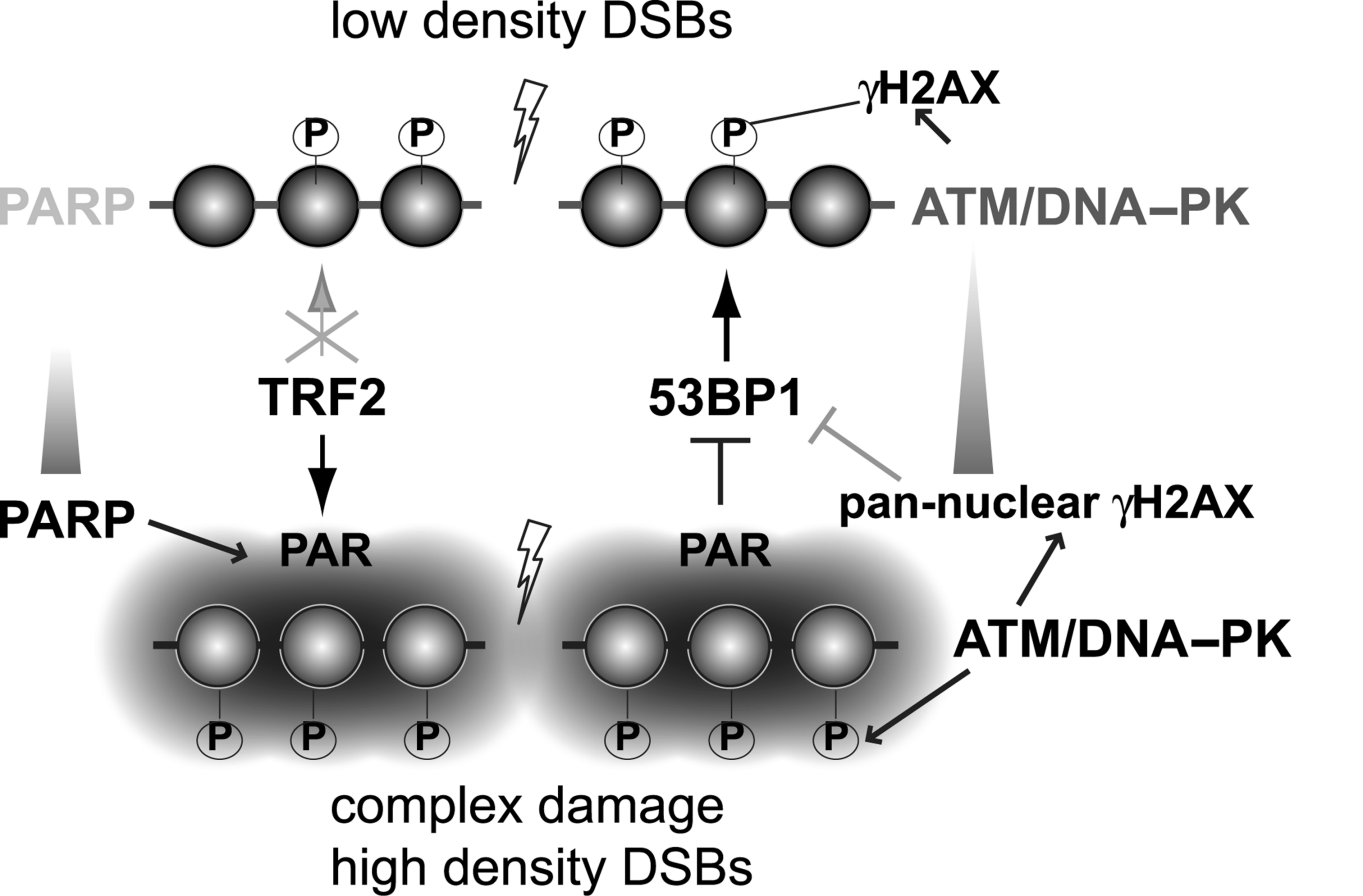
Regulation of 53BP1 and TRF2 recruitment to low density DSBs and complex damage with high-density DSBs. For relatively simple DSBs at low density, efficient 53BP1 recruitment occurs while no significant PARP activation and TRF2 accumulation are observed. In contrast, complex damage with high-density strand breaks induces robust PARP activation and promotes TRF2 recruitment while inhibiting 53BP1 recruitment. ATM/DNA–PK-dependent pan-nuclear γH2AX induced by high-density DSBs also indirectly reduces 53BP1 clustering at damage sites.

We previously demonstrated that the femtosecond (fs) NIR laser can precisely generate DNA lesions such as SSBs and DSBs within a submicron focal spot in the nucleus without damaging the cell membrane (38–40). This approach allows monitoring subcellular DDR and associated repair factor assembly processes *in vivo*. However, in many laser microirradiation studies (using an ultra-short fs NIR laser or other laser systems) to analyze DDR, complete irradiation parameters (i.e. pulse duration, pulse repetition rate, energy of each laser pulse (nJ), focal spot peak irradiance (W/cm^2^), exposure time and laser wavelength) are frequently not provided (4). Furthermore, the effects of changing irradiation conditions on the observed DDR are often not evaluated. We previously compared different wavelength laser systems to study cellular responses to DNA damage in mammalian cells (5). In the current study, we performed detailed titration analysis of laser input powers using two different fs NIR laser systems (Mira-900 and Meta), and defined the distinct threshold power and energy for the recruitment of TRF2 and 53BP1 whose laser-damage site recruitment conditions were previously controversial. Our results reveal that a higher input laser power (irradiance) results in an increase of strand breaks and complex DNA damage, and is accompanied by robust activation of ATM/DNA–PK and PARP signaling. We found that 53BP1 and TRF2 recruitment to damage sites is critically dictated by these signals, rather than amount and/or complexity of the damage per se (Figure 7). Importantly, our results highlight both positive and negative roles of PARP signaling in the regulation of repair factor assembly, and thus, choice of repair pathway.

### PARP inhibition stimulates 53BP1 recruitment to damage sites

Our results demonstrate that strong ATM/DNA–PK and PARP responses induced by high input-power damage are inhibitory to 53BP1 recruitment. Though Chk2 may also play a role, ATM/DNA–PK-dependent spreading of γH2AX followed by dispersion of MDC1 most likely contributes to reduced 53BP1 association at damage sites. PARP inhibition also partially restored 53BP1 recruitment without affecting MDC1, and co-inhibition of ATM/DNA–PK and PARP almost completely restored 53BP1 recruitment to both high- and low-input-power damage sites in the same nucleus, strongly suggesting that the inhibitory effects of two signal responses are independent of each other. Indeed, even with a low input-power damage alone that does not induce pan-nuclear γH2AX, Pi, but not Ai+Di, significantly enhanced immediate early 53BP1 recruitment. Forced accumulation of PAR by PARG inhibition effectively suppressed normally robust 53BP1 recruitment to low input-power damage sites. Thus, the strength of PAR signal primarily regulates the initial 53BP1 accumulation at damage sites.

How 53BP1 recruitment to damage sites is inhibited by PARP signaling is currently unclear. It is possible that local accumulation of PAR chains at damage sites may cause steric hindrance and interfere with 53BP1 binding to methylated H4K20 and ubiquitylated H2A K15. PAR was recently shown to nucleate accumulation of intrinsically disordered proteins (IDPs) to damage sites, which may antagonize 53BP1 recruitment (55). PARP activity was also shown to be important for the rapid SUV39h1 targeting to damage sites and a transient H3K9me3 heterochromatinization at DSB sites, which is necessary for the subsequent recruitment of Tip60 acetyltransferase (56,57). Since Tip60-dependent histone H4 acetylation was shown to inhibit 53BP1 recruitment (58), it is possible that the observed inhibition of 53BP1 by PARP activity may be indirectly through upregulation of H4 acetylation.

It is interesting to speculate that restricting 53BP1 recruitment may affect DNA repair pathway choice. For example, PAR-dependent transient inhibition of 53BP1 may promote efficient BER in the context of complex DNA damage. Furthermore, 53BP1 promotes NHEJ by suppressing the end-resection by CtIP necessary for HR while BRCA1 promotes HR (15–19). PARP inhibitors were shown to increase DNA damage sensitivity of BRCA1 mutant cancer cells (59,60), which appears to be driven by NHEJ activation rather than BER inhibition (61). This sensitivity was alleviated by depletion of 53BP1 (15,62,63). It was thought that PARP normally suppresses NHEJ, which is hyperactivated by PARP inhibitor treatment in HR-defective cells, and that NHEJ suppression by 53BP1 inactivation restores the balance between the two repair pathways (61). While the antagonistic roles of PARP1 and Ku was suggested (64,65), our results indicate that 53BP1 recruitment itself is suppressed by PARP, providing new insight into the action of PARP inhibitors.

### Rapid and transient TRF2 recruitment to damage sites is PARP-dependent

TRF2 recruitment to damage sites has long been controversial, but the consensus was that higher power laser damage is required for its efficient recruitment to damage sites (11,29,30). We found that TRF2 recruitment is dependent on PAR, which appears to be the sensor for high dose complex DNA damage, providing the molecular explanation for the observed differential recruitment of TRF2. A PARP family member Tankyrase localizes to telomeres, and interacts with and affects telomere association of TRF1, but not TRF2 (66,67). TRF2 was shown to interact with PARP1 (53) and promote the recruitment of PARP1 to eroded telomeres (53,54). TRF2 targeting to telomeres is not PAR-dependent, and PARP activity was inhibitory to TRF2 binding to telomere DNA *in vitro* (53,54). At DNA damage sites, TRF2 is not required for PARP1 recruitment, and PARP1 without PARP activity was not sufficient for TRF2 recruitment. Thus, the mechanism of precipitous TRF2 recruitment to damage sites is distinct from that for telomere targeting. Since TRF2 dissociates from damage sites before PAR signal disappears, however, PARP may subsequently destabilize TRF2 association with damaged DNA as was shown with telomere DNA (53,54).

### PARP-regulated chromatin factor assembly at damage sites

A number of factors involved in DNA repair, in particular chromatin regulators, were found to be recruited to damage sites in a PAR-dependent manner, including ALC1, the NuRD complex, the PcG complexes, macroH2A and more recently KDM4D (2,68). ALC1 is a member of the SNF2 superfamily of ATPases (69,70). The NuRD complex contains chromatin remodeling and histone deacetylase and demethylase activities and functions in transcriptional repression (71–74). PcG protein-containing complexes PRC1 and PRC2 as well as macroH2A are also involved in epigenetic gene silencing (71,75). KDM4 is a histone demethylase specific for H3 lysine 9 methylation (H3K9me) (68). These factors were all found to rapidly cluster to damage sites in a PAR-dependent manner, presumably contributing to the rapid chromatin organization at damage sites, which is important for the subsequent repair process. While the exact role of TRF2 in DDR remains to be determined, our results raise the possibility that TRF2 belongs to the same class of DDR proteins that are recruited to damage sites via PARP signaling. We speculate that these chromatin regulators may be preferentially recruited to, and together, facilitate the resolution of DNA lesions consisting of complex damage. This is in a stark contrast to 53BP1, which prefers DSBs that do not trigger a strong PARP response. Taken together, our study reveals both positive and negative roles for PARP signaling in DDR factor assembly at damage sites, which may be critical for proper resolution of different types of DNA damage.

### The effects of different laser dose on the mechanisms of DNA damage induction

The interaction of the laser light with living tissue/cells may trigger various physical and chemical processes that can potentially produce structural and/or biochemical damage. These may be either thermal or non-thermal processes, depending on the absorption properties of the biological specimen as well as the laser irradiation parameters (76). In general, the potential mechanisms for laser-induced damage on biological structures include: (i) direct heating of the sample produced by linear or two-photon absorption processes, (ii) generation of large thermo-elastic stresses, (iii) photochemical processes generated by the linear and two-photon absorption, including crosslinking damage and production of the cytotoxic agents such as free radicals and reactive oxygen species (ROS), and (iv) thermal, mechanical, and chemical processes emanating from optical breakdown (plasma formation) produced by a combination of multiphoton ionization and cascade ionization processes. All of these mechanisms may contribute to laser-induced damage phenomena when using rapidly pulsing lasers that deliver a high number of pulses in a single irradiation event. It is likely that the first or first several laser pulses alter the absorption properties of the biological specimen resulting in a different interaction of the remaining pulses of the pulse train with the target structure. This secondary laser pulse-target interaction may be thermal whereas the interaction of the first (or first several) pulses may cause structural alteration through multiphoton or plasma-induced mechanisms (39). Which laser damage mechanisms occur, either alone, or in combination, may vary depending upon the laser parameters used, such as wavelength, pulse duration, pulse frequency (repetition rate), energy density (J/cm^2^) and the irradiance (W/cm^2^). Therefore, in order to understand an observed laser-induced damage process, it is important to consider the laser parameters used, and particularly, to take into account differences in these parameters when different laser systems are used.

The application of the ultra-short fs NIR lasers enables precise site-specific nano-processing and cellular nanosurgery with low pulse energies, and thereby minimal subsequent collateral destructive effects beyond the absorption/irradiation site. Compared to the nanosecond and picosecond lasers, the damage production of the fs NIR lasers are mainly due to multiphoton effects such as multiphoton ionization and plasma formation, with minimum effects from shockwave and cavitation bubble formation (77,78).

Although the absorption coefficient of the biological structures (chromosomes) and the heating effects at NIR wavelength are not significant compared to the shorter wavelengths such as in the UV range (79), the cumulative heating effects are important while using lasers with very high repetition pulse rates. The significance of the cumulative heating can be investigated by considering the photo-thermal confinement in the laser focal spot. For this purpose, the thermal diffusion time constant is defined as: *T*_d_ = 0.124*λ*^2^/*k* (NA)^2^; where *k* is the thermal diffusivity of the surrounding medium which, in our study, is assumed to be that of water (1.4 × 10^−7^ m^2^/s); *λ* is the wavelength of the laser, and NA is the numerical aperture of the objective (80). In our Mira-900 system, with the *λ* and NA values of 800 nm and 1.4, respectively, the corresponding value of *T*_d_ would be ≈289 ns. Similarly, for the Meta system the corresponding value of *T*_d_ is ≈319 ns with the *λ* and NA values of 780 nm and 1.3, respectively. Since in both laser systems the *T*_d_ values are greater than the laser pulse duration, i.e. 289 ns versus 200 fs in Mira-900 system, and 319 ns versus 140 fs in the Meta system, photo-thermal confinement occurs in the focal volume during microirradiation. Under this condition, the time needed for the absorption of the laser light and the subsequent dissipation of the generated heat in the focal volume is much greater than the duration of laser pulses. Therefore, it is likely that generated heat is accumulated at the high pulse repetition rates used.

In our study, the way in which the laser pulses are delivered is different for the two laser systems, and as a result, the accumulation of the thermal energy is different. In the Mira-900 system, the laser irradiated a region of diffraction-limited size for 10 ms duration at 76 MHz with 200 fs micropulses. This was followed by a 100 ms delay in irradiation before the laser beam was moved to the next position by a laser scanning mirror and the next 10 ms laser irradiation occurred. In contrast, the Meta system continuously scanned the region of interest with a laser that delivered the 80 MHz beam containing 140 fs micropulses. In the Mira-900 system, given the 100 ms time delay between the macropulses, there is a significant potential for the relaxation of the photo-thermal confinement effects and the subsequent dissipation of the accumulated heat. Such a time delay is not present in the Meta system. This may explain why less amounts of total energy delivered and smaller peak irradiances are needed in the Meta system compared to the Mira-900 system, in order to observe similar biological damage responses. To estimate the amount of cooling between the macropulses in the Mira-900 system, we assume the interphase chromosome as a thin slab of diameter *d* (1 μm in our study) and that it is uniformly heated by the laser irradiation. Under those conditions, the time-dependent change of the temperature at the center of the chromosome sheet can be estimated as: $\frac{{\Delta T(t)}}{{\Delta T(t = 0)}} = 1 - \exp \left( { - \frac{{d^2 }}{{4kt}}} \right)$. Assuming the Δ*T*(*t* = 0) as the temperature of the chromosomes at the end of a 10 ms macropulse exposure, the fraction of the heat retained after a 100 ms (*t*) delay before the next 10 ms macropulse exposure, would be: $\frac{{\Delta T(100ms)}}{{\Delta T(t = 0)}}$ ≈1.78 × 10^−5^. Therefore, with the Mira-900 system, only a very small percent of the generated heat (0.0017%) will be retained for the next macropulse. Since the cooling mechanism is not present in the Meta system, its shorter pulse (140 fs versus 200 fs) duration and higher repetition rate also may contribute to higher temperature rise and heat accumulation as well as possible damage by other physical mechanisms. With respect to the pulse duration, in a previous study with a 75 MHz fs NIR laser, it was demonstrated that the damage (induction of transient pores) of stem cell membranes required much less energy (≈16 times) with 12 fs laser pulses compared to 250 fs pulses (77).

In addition to the cumulative temperature rise, the application of the ultra-short fs laser pulses may lead to production of thermally induced (thermo-elastic) stresses (81). These thermal stresses may cause structural damage. Their significance can be determined by the stress relaxation factor (*τ*_m_) which is defined as the ratio of the laser pulse duration (*t*_p_) to the time necessary for the stress waves to propagate through the heated structure (*t*_s_). With the parameter *t*_s_ defined as the ratio of the characteristic thickness of the sample (*d*) to the speed of sound (*c*_s_ = 2600 m/s), *τ*_m_ = *t*_p_.*c*_s_/*d*. Assuming *d* as 1 μm, *τ*_m_ would be ≈3.6 × 10^−4^ and 5.2 × 10^−4^ for the Mira-900 and the Meta system, respectively. The values of *τ*_m_ < 1 imply that the time needed for the thermo-elastic stresses to be dissipated is greater than the duration of each laser pulse, therefore the thermo-elastic stress confinement would occur in the focal spot. Previous studies estimated the magnitude of the stress produced by an individual fs NIR pulse to be as small as ≈0.014 MPa (80) which, in our study, is unlikely to produce the observed DNA damage. In addition, the peak irradiances used with both lasers in our study are in the range of 10^10^–10^11^ W/cm^2^. This is less than the ≈5 × 10^12^ W/cm^2^ reported as the threshold for thermo-elastic stress confinement in the fs NIR beam (80). Therefore, despite the presence of the thermo-elastic stress confinement, their magnitude may not be sufficient to induce the DNA damage observed in our study. In addition, given the 100 ms delay between the consecutive macropulses in the Mira-900 system their contribution is likely less with the Mira-900 system than with the Meta system.

Multiphoton absorption can drive chemical reactions leading to production of highly reactive cytotoxic agents including free radicals and ROS (82). In addition to these species causing indirect damage, depending on the wavelength of the laser, multiphoton absorption may also cause photo-ablation effects and dissociation of molecular bonds that occur with high-energy UV photons. Such UV effects (266 nm) are also generated by a two-photon absorption particularly with 532 nm ns pulsed lasers (83). Studies with the fs lasers suggested significant nonlinear interactions with biological materials (84,85). Using fs NIR lasers, the DNA damage is likely facilitated by non-linear absorption by the DNA/chromatin structure via a two- or three-order multiphoton process (86). The threshold peak irradiance previously estimated for the photochemical damage in fs NIR lasers is ≈0.26 × 10^12^ W/cm^2^ (80). This threshold is within the range we used with the Mira-900 laser but is higher than the range used with the Meta system.

In addition to the absorption of the photons by the target, another key factor in determining the damage mechanism is the irradiance in the focal volume, which can be very high with ultra-short laser pulses. With fs NIR laser pulses, at irradiances higher than a threshold, i.e. ≈6 × 10^12^ W/cm^2^ for a transparent media (80), optical breakdown or laser-induced plasma formation may occur. This results in creation of quasi-free electrons in the laser focal volume that can damage the biological structure via chemical decomposition (bond breaking) and multiphoton induced chemistry. Thermal (temperature rise), mechanical (creation and propagation of shockwaves and cavitation bubble dynamics), and photochemical (combination of multiphoton and cascade ionization) processes can evolve from the plasma formation which may cause significant damage to the DNA/chromatin structure.

Although the peak irradiances used in our study (for both of the laser systems) are smaller than the plasma formation threshold, pulse energies below the plasma threshold using picosecond and femtosecond lasers can create significant damage to biological structures through formation of low-density plasmas (regions with free electron density below 10^21^ e^−^/cm^3^) (87–90). Studies have demonstrated that regions with free electron densities as small as 10^15^ e^−^/cm^3^ can generate significant thermal, mechanical, and chemical damage leading to intracellular ablation and dissection (89). Such free electron densities can be produced by pulse energies of only 5% of the threshold energy for plasma formation. In a specific study on DNA, irradiation of plasmid DNA with a monochromatic low energy electron beam resulted in significant genotoxic damage through rapid decay of transient molecular resonances localized on the DNA molecule (91). With the electron energies well below the ionization threshold of DNA, substantial SSB and DSB breaks were generated by the secondary electrons and their ionic and radical reaction products (secondary electrons are generated by primary ionizing radiation) (91). Taken together, low-density plasma regions and low-energy free electrons can affect the DNA/chromatin structure.

## CONCLUSION

Our laser power titration experiments reveal distinct laser power thresholds required for damage site association of 53BP1 and TRF2, which is determined by differential ATM/DNA–PK and PARP activation reflecting changes in the number of DSBs and the complexity of the DNA damage. Our results emphasize that careful attention must be given to the titration of the laser irradiance and energy density in the focal spot as subtle changes of the laser dose affects the types and amounts of induced DNA damage and the subsequent DDR. Once titrated, it is possible to obtain comparable results using different NIR laser systems. Our study highlights the positive and negative roles of the PAR response at damage sites that fine-tune how the cell processes different amounts and complexities of DNA lesions.

## Supplementary Material

SUPPLEMENTARY DATA
