# Supplementary material for: Femtosecond near-infrared laser microirradiation reveals a crucial role for PARP signaling on factor assemblies at DNA damage sites
Source: Nucleic Acids Res. 2015 Sep 30;44(3):e27. doi: 10.1093/nar/gkv976 (PMC4756852; doi:10.1093/nar/gkv976)
Supplement: SUPPLEMENTARY DATA [file supp_44_3_e27__index.html]

Femtosecond near-infrared laser microirradiation reveals a crucial role for PARP signaling on factor assemblies at DNA damage sites — Femtosecond near-infrared laser microirradiation reveals a crucial role for PARP signaling on factor assemblies at DNA damage sites — SUPPLEMENTARY DATA 

# Femtosecond near-infrared laser microirradiation reveals a crucial role for PARP signaling on factor assemblies at DNA damage sites

## SUPPLEMENTARY DATA

- SUPPLEMENTARY DATA
