# Supplementary material for: Femtosecond near-infrared laser microirradiation reveals a crucial role for PARP signaling on factor assemblies at DNA damage sites
Source: Nucleic Acids Res. 2015 Sep 30;44(3):e27. doi: 10.1093/nar/gkv976 (PMC4756852; doi:10.1093/nar/gkv976)
Supplement: SUPPLEMENTARY DATA [file supp_gkv976_nar-01365-met-k-2015-File009.pdf]

Supplemental Figure S1

A

		damaged cell	observation period	survived
Meta laser	40%	7	8hrs	7
	30%	8	8hrs	8
	20%	9	8hrs	9
	5-10%	12	8hrs	12
Mira-900 laser	100mW	8	7hrs	8
	90mW	9	7hrs	9
	25mW	8	7hrs	8
	20mW	8	7hrs	8

B

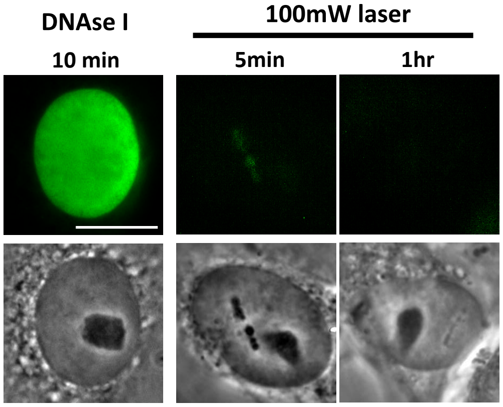

Supplemental Figure S1.
At single cell level, the viability of the PtK2 cells, which were microirradiated at different conditions as indicated, were checked 7-8 hrs after DNA damage induction **(A)**, particularly cells irradiated with high input-power laser (100mW) were subjected to TUNEL assay **(B)**. TUNEL signals were shown at DNA damage sites (but not the whole nucleus) 5 min post irradiation (p.i.), and were even weaker, if there were some, after 1hr. DNase I (3000U/ml, 10min)-treated cell served as positive control.

Supplemental Figure S2

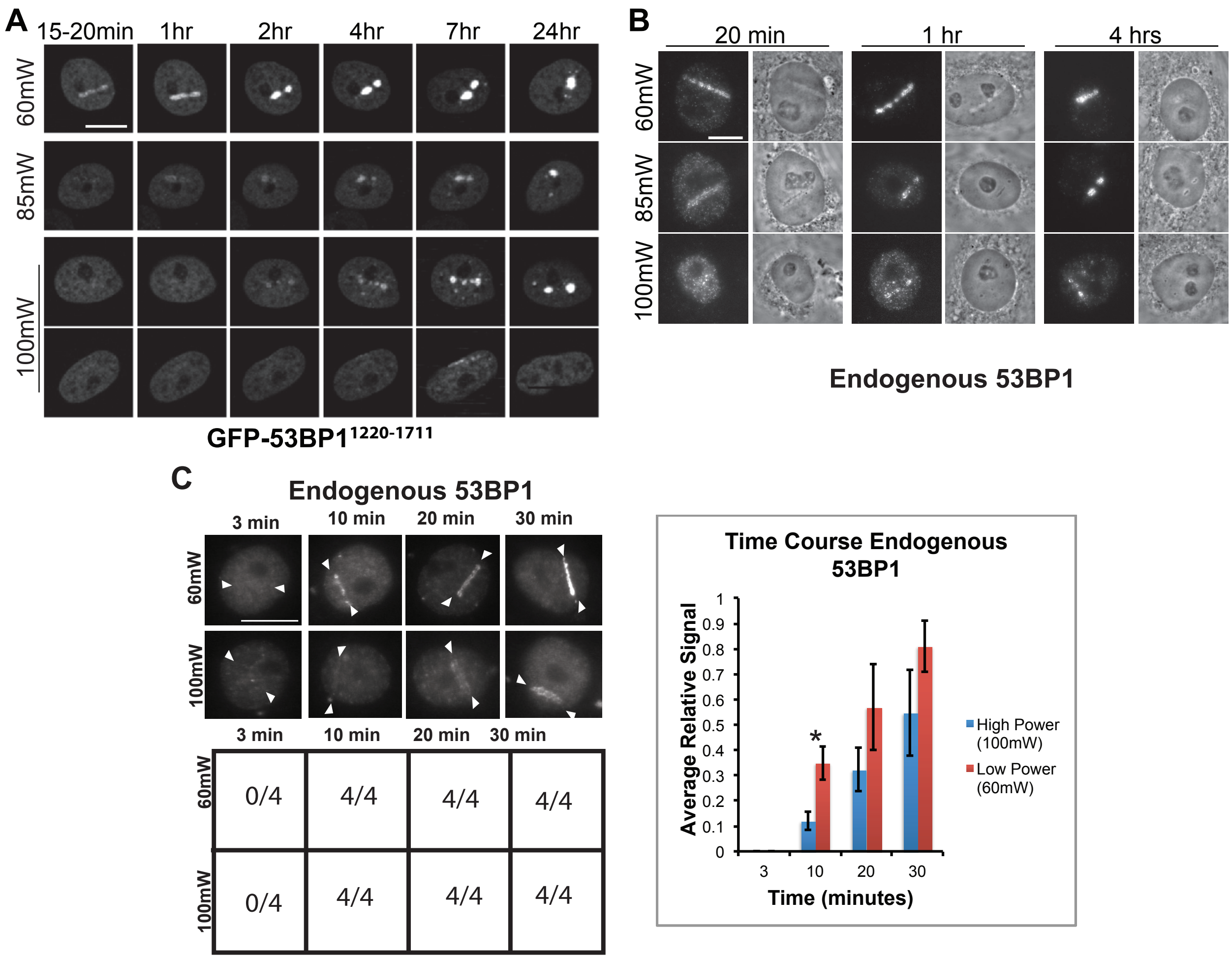

Supplemental Figure S2.
(A) Live-cell time course analysis of EGFP-53BP1$^{1220\text{-}1711}$ recruitment to the laser-induced damage sites. EGFP-53BP1$^{1220\text{-}1711}$ PtK2 cells were damaged with the input powers of 60 mW, 85 mW, or 100 mW produced by a NIR laser source. The cells were monitored up to 24 hrs and fluorescent confocal images were taken at indicated time points p.i. (N=5 for each input power). For 100 mW, two and three cells exhibited the recruitment patterns shown in the upper and lower panels, respectively. Scale bar =10 µm.
(B) Immunofluorescent staining of the endogenous 53BP1 recruitment to the laser-induced damage sites. Non-transfected PtK2 cells were damaged as in (A), fixed at 20 min, 1 hr or 4 hrs after irradiation as indicated, and stained with antibody specific for 53BP1. N=3 for each condition, which exhibited similar recruitment patterns. Scale bar =10 µm.
(C) Time course analysis of the endogenous 53BP1 following 60mW and 100mW damage in the Mira-900 system. PtK2 Parental cells were fixed at specific time points p.i. as indicated at the top. Number of cells with positive 53BP1 at damage sites and number of cells tested are shown in the box below. Right: quantitative analysis of average 53BP1 recruitment at each time point. Fold increase in the relative fluorescence intensity of 53BP1 at laser-microirradiated sites compared with the nucleoplasm is shown.

Supplemental Figure S3

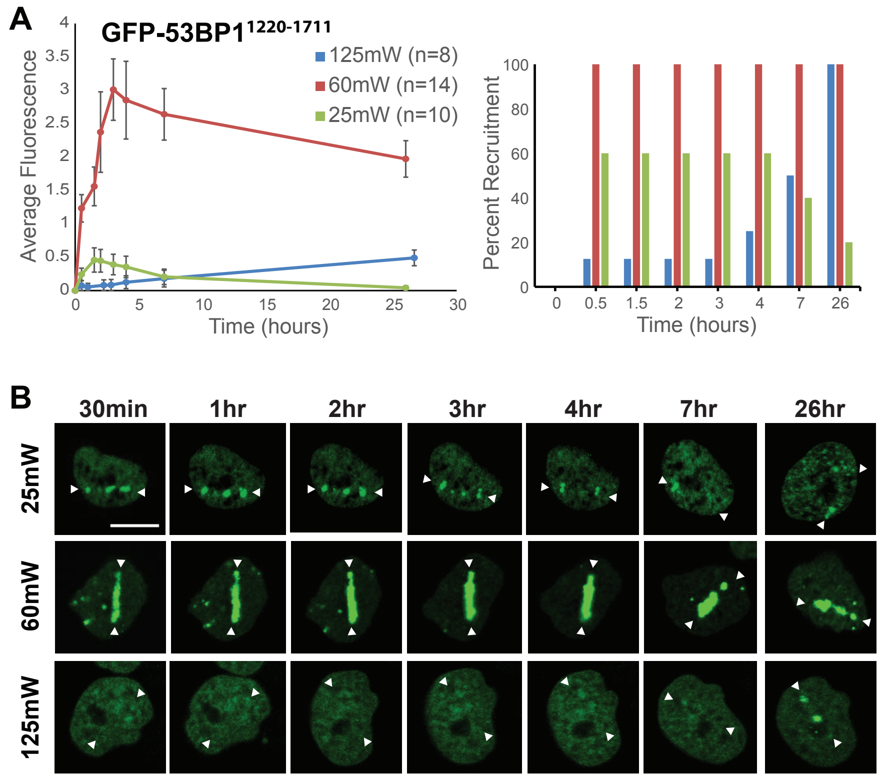

Supplemental Figure S3.

(A) Ptk2 cells stably expressing EGFP-53BP1$^{1220\text{-}1711}$ were damaged with input powers of 25mW, 60mW, and 125mW using the NIR laser. Cells were monitored up to 26 hrs p.i. and the fluorescence signals normalized against the nuclear background signals in the same cells were averaged for each power (Left). Percentage of damaged cells with positive EGFP-53BP1$^{1220\text{-}1711}$ recruitment at each time point is shown (Right).

(B) Representative EGFP-53BP1$^{1220\text{-}1711}$ cells from (A) are shown.

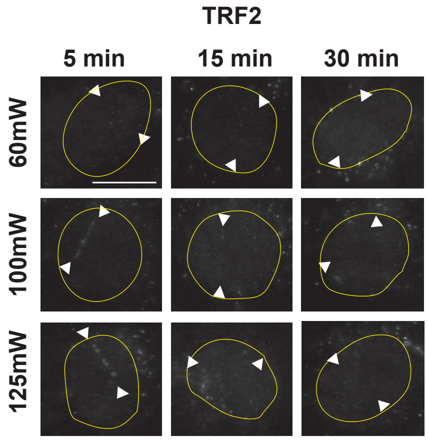

	5 min	15 min	30 min
60mW	0/12	0/12	0/12
100mW	7/12	1/12	0/12
125mW	10/12	0/12	0/12

Supplemental Figure S4.
Immunofluorescent staining of the endogenous TRF2 in PtK2 cells presensitized with Hoechst and damaged with three different input power dosages. Cells were fixed at time points indicated at the top. Number of cells tested and number of cells in which TRF2 recruitment was observed are shown in the box.

Supplemental Figure S5

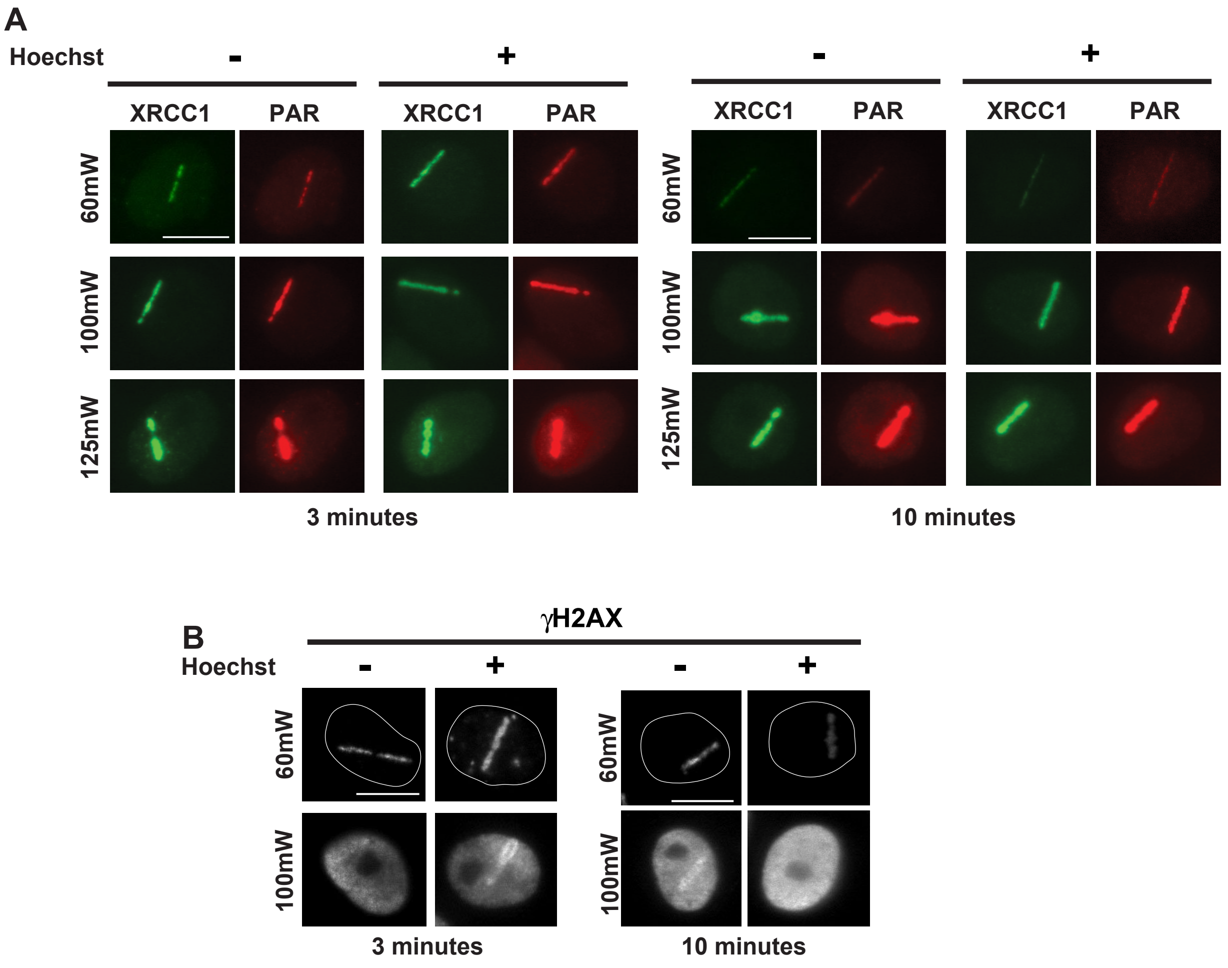

Supplemental Figure S5.
PtK2 cells were damaged with indicated input laser power with and without presensitization with Hoechst. Cells were then fixed at 3 or 10 min p.i. and were stained with antibodies specific for XRCC1 and PAR (N=5, consistent results) **(A)** and γH2AX (N=10, consistent results) **(B)**.

Supplemental Figure S6

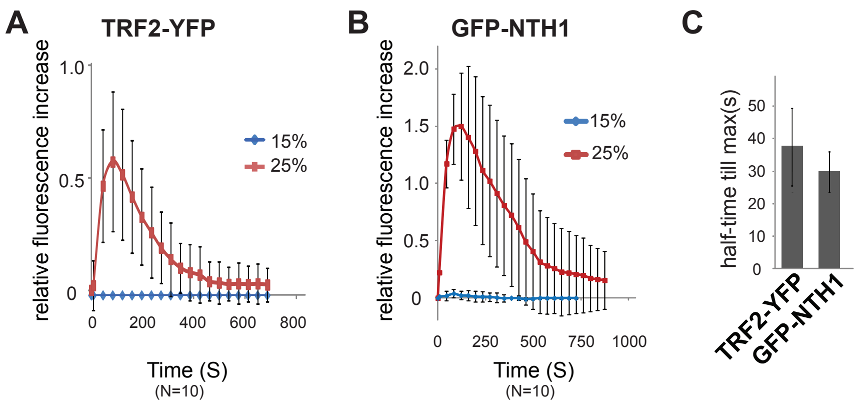

Supplemental Figure S6.
(A) Live-cell time course analysis of TRF2-YFP recruitment to the laser-induced damage sites. Quantification of relative fluorescent signal increase of TRF2-YFP in PtK2 cells using 15% (blue) or 25% (red) input power in the Meta system is shown. (N=10 each).
(B) Similar analysis of GFP-NTH1 DNA glycosylase as in (A) (N=10 each).
(C) Half-time till maximum accumulation for each treatment is also shown.

Supplemental Figure S7

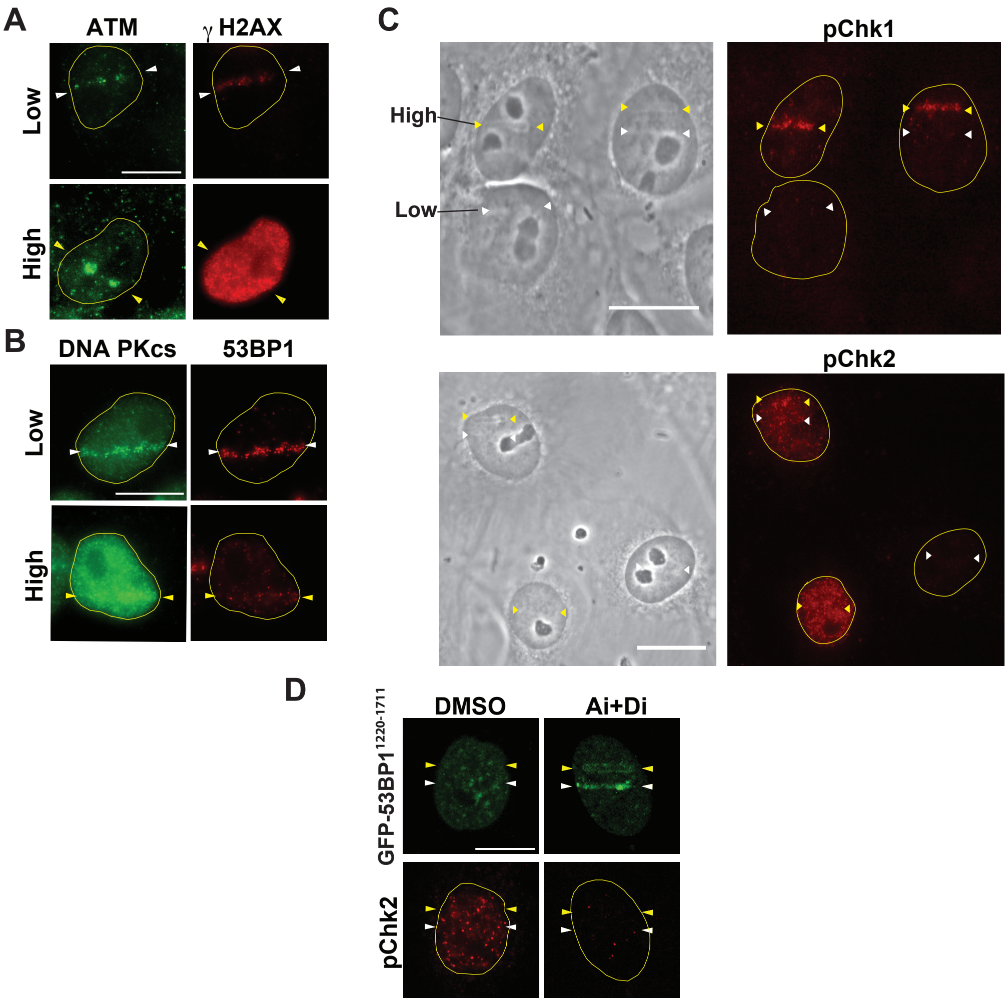

Supplemental Figure S7.
(A) Immunofluorescent staining of HeLa cells damaged with either low (15%, indicated by white arrows) or high (25%, indicated by yellow arrows) input power using the Meta system. Cells were fixed at 1 hr p.i., permeabilized and immunostained with antibodies specific for ATM and γH2AX as indicated. Consistent results were obtained with all cells examined (N=5). Scale bar =10 μm.
(B) Immunofluorescent staining of HeLa cells damaged, fixed and permeabilized in the same way as in (A) using antibodies specific for DNA-PK and 53BP1. Consistent results were obtained with all cells examined (N=5). Scale bar =10 μm.
(C) PtK2 cells were irradiated with either single low, single high, or both low/high input power conditions using the Meta system. Cells were then fixed after 20-30 min p.i. and were stained using antibodies specific for pChk1 or pChk2 as indicated. Consistent results were obtained with all cells examined (N=6 each). Scale bar=30μm.
(D) PtK2 cells stably expressing EGFP-53BP1$^{1220-1711}$ with both low and high input-power damage sites were treated with DMSO or Ai+Di, and were stained with antibody specific for pChk2. Scale bar =10 μm. Consistent results were obtained with all cells examined (N=10 each).

Supplemental Figure S8

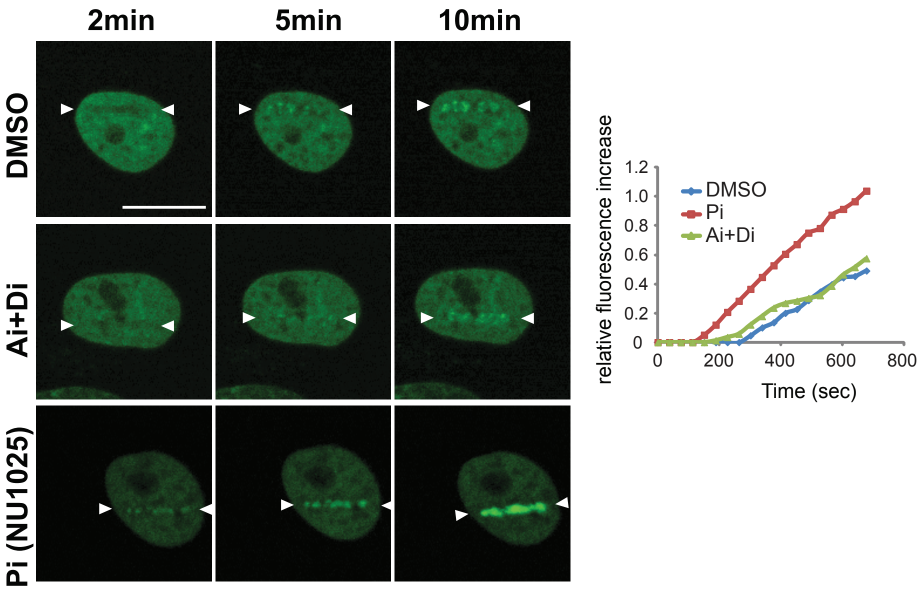

Supplemental Figure S8.

The effect of Pi (NU-1025) on the recruitment of EGFP-53BP1$^{1220\text{-}1711}$ in response to low input-power damage. Live-cell time course analysis of EGFP-53BP1$^{1220\text{-}1711}$ recruitment to the laser-induced damage sites in PtK2 cells treated with DMSO, Pi or Ai+Di using 15% input power in the Meta system. Cell images were taken at the indicated time points p.i. Quantification of relative fluorescent signal increase for each treatment is shown. First 10 min of accumulation is shown while the damage site accumulation of EGFP-53BP1$^{1220\text{-}1711}$ normally become prominent after 15-20 min p.i. in control cells (DMSO). Consistent results were obtained with all cells examined (N=5 each). Scale bar =10 μm.

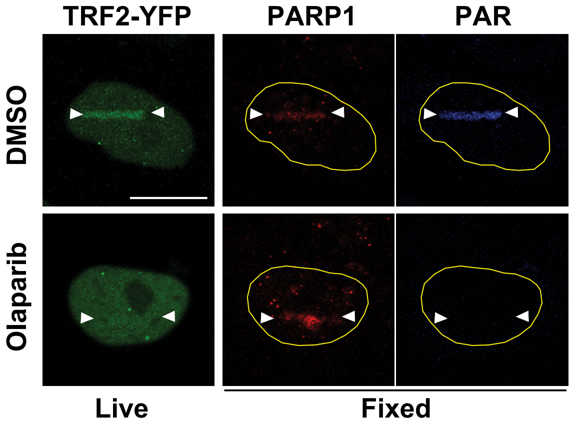

Supplemental Figure S9.
PtK2 cells stably expressing TRF2-YFP were treated with DMSO or PARP inhibitor (olaparib) and damaged with 25% input Meta laser power. Live cell image of TRF2-YFP was taken at 1-2 min p.i. at the peak of its recruitment. Cells were then fixed at 15 min p.i. and co-stained with antibodies specific for PARP1 and PAR (N=3 each with consistent results). Scale bar =10 μm.

Supplemental Fig. S10

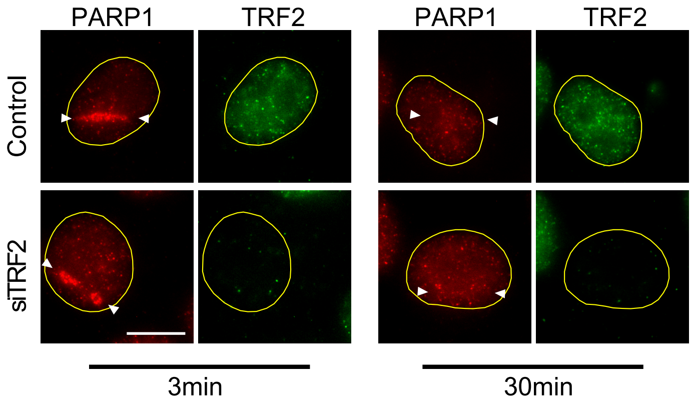

Supplemental Figure S10.
HeLa cells were transfected with TRF2 or control siRNA (SI00742630 and 1022076; Qiagen, Valencia, CA) (1) as described previously (2) for 48 hrs and were fixed and stained with antibodies specific for PARP1 and TRF2 as indicated at the top at 3 min and 30 min p.i. with 25% input power in the Meta system (N=8 each with consistent results). Scale bar =10 µm.

1. Mao, Z., Seluanov, A., Jiang, Y. and Gorbunova, V. (2007) TRF2 is required for repair of nontelomeric DNA double-strand breaks by homologous recombination. Proc. Natl. Acad. Sci., 104, 13068-13073.
2. Kong, X., Ball, A.R., Jr., Pham, H.X., Zeng, W., Chen, H.Y., Schmiesing, J.A., Kim, J.S., Berns, M. and Yokomori, K. (2014) Distinct functions of human cohesin-SA1 and cohesin-SA2 in double-strand break repair. Mol. Cell Biol., 34, 685-698.
